# Supplementary material for: Gene Regulation by Antitumor miR-204-5p in Pancreatic Ductal Adenocarcinoma: The Clinical Significance of Direct RACGAP1 Regulation
Source: Cancers (Basel). 2019 Mar 7;11(3):327. doi: 10.3390/cancers11030327 (PMC6468488; doi:10.3390/cancers11030327)
Supplement: Supplementary file 1 [file cancers-11-00327-s001.pdf]

Supplementary Materials: Gene regulation by antitumor *miR-204-5p* in pancreatic ductal adenocarcinoma: the clinical significance of direct RACGAP1 regulation

Muhammad Khalid, Tetsuya Idichi, Naohiko Seki, Masumi Wada, Yasutaka Yamada, Haruhi Fukuhisa, Hiroko Toda, Yoshiaki Kita, Yota Kawasaki, Kiyonori Tanoue, Hiroshi Kurahara, Yuko Mataki, Kosei Maemura and Shoji Natsugoe

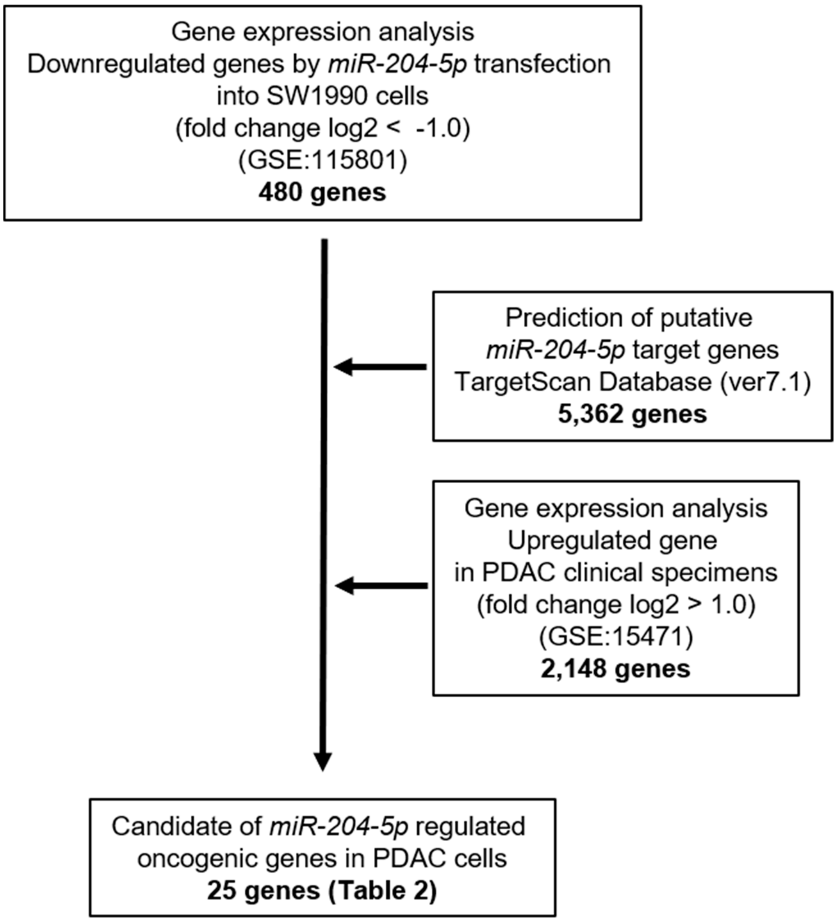

Figure S1. Strategy for identification of putative genes regulated by *miR-204-5p* in PDAC cells. Our previous gene expression analyses showed that a total of 480 genes were downregulated by *miR-204-5p* transfection into SW1990 cells. The gene expression data were deposited in the GEO database (accession number: GSE115801). Among these genes, 25 were also upregulated in PDAC clinical specimens (GEO accession number: GSE15471) and also contain putative *miR-204-5p* binding sites in their 3'untranslated regions (according to the TargetScan database).

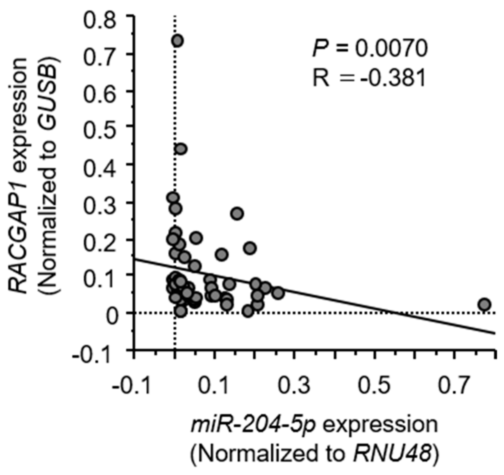

Figure S2. Expression levels of *RACGAP1* and *miR-204-5p* were negatively correlated.

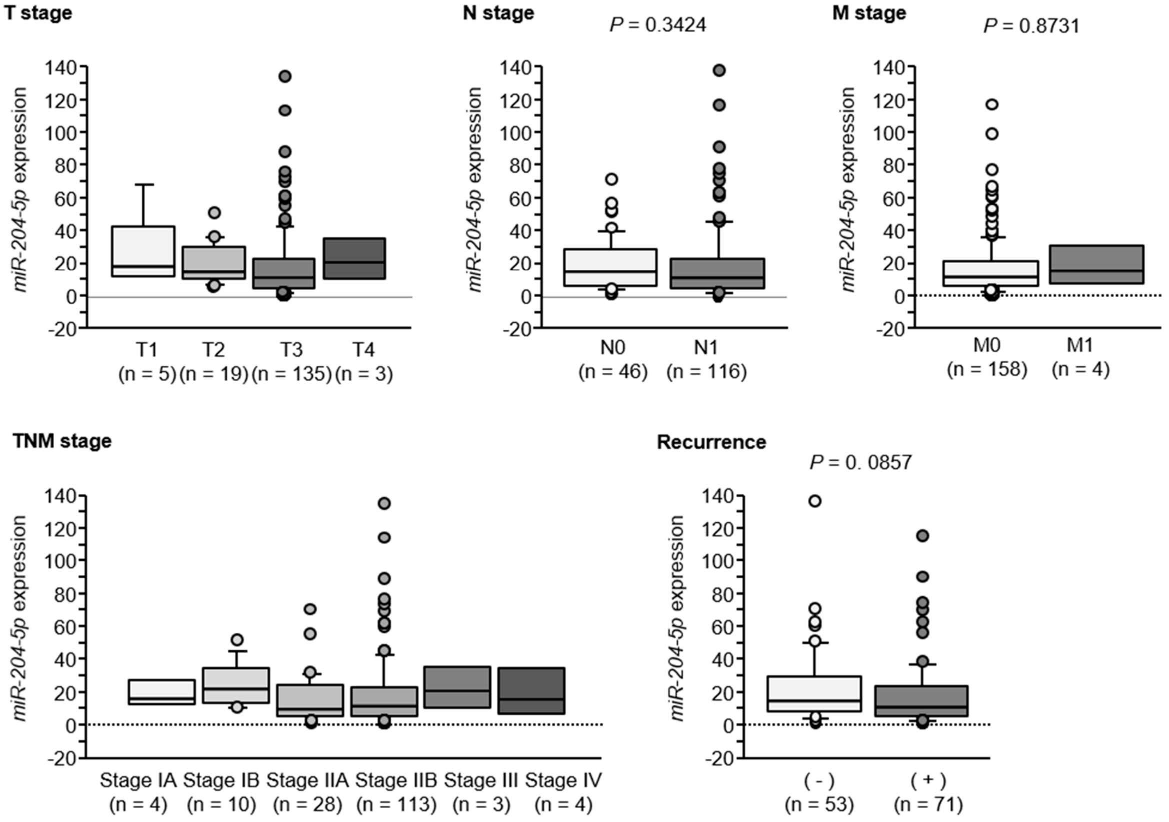

Figure S3. Analysis of clinicopathological factors related to *miR-204-5p* expression in TCGA database from cBioportal.

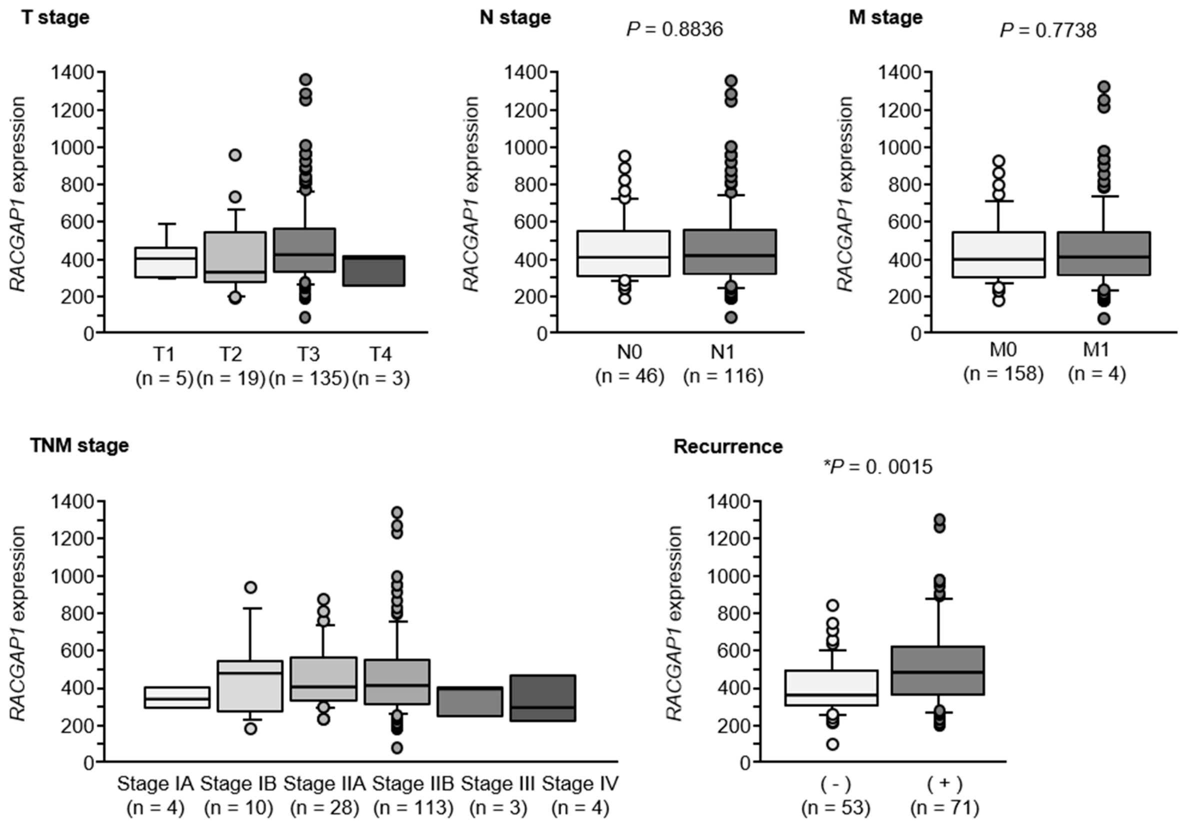

Figure S4. Analysis of clinicopathological factors related to *RACGAP1* expression in TCGA database from cBioportal.

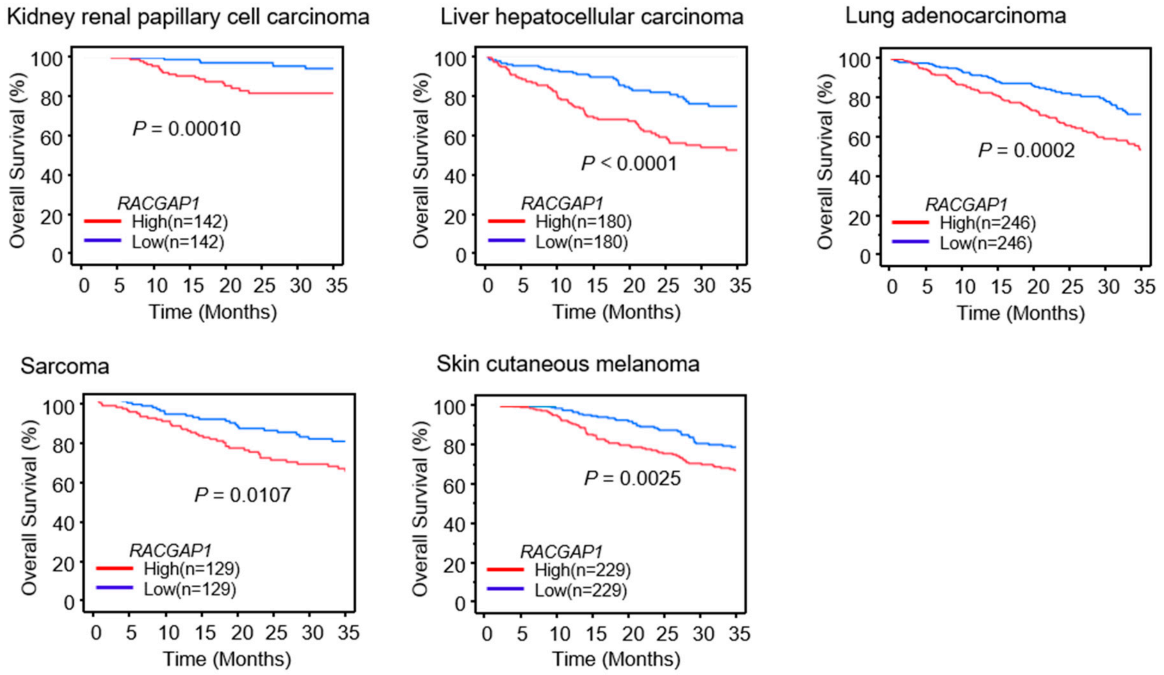

Figure S5. TCGA database analyses showed that high expression of *RACGAP1* was closely associated with poor prognosis (3-year OS) of several cancers, e.g., kidney renal papillary cell carcinoma, hepatocellular carcinoma, lung adenocarcinoma, sarcoma and skin cutaneous melanoma.

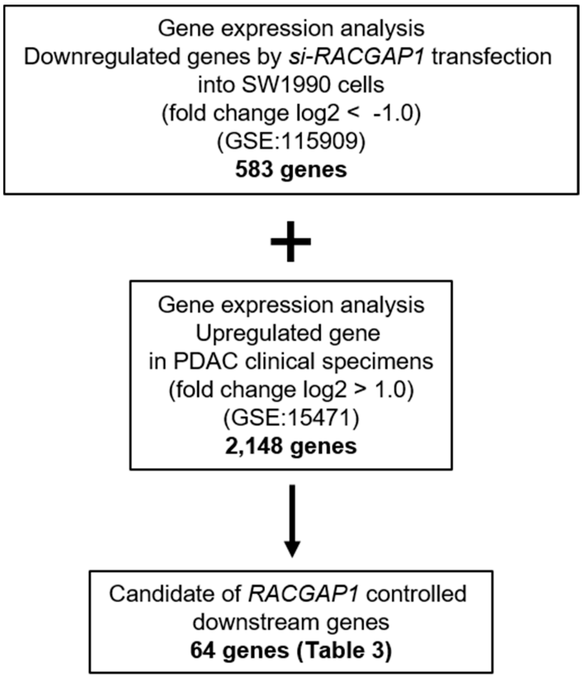

Figure S6. Strategy for identification of putative genes regulated by *RACGAP1* in PDAC cells. Our previous gene expression analyses showed that a total of 583 genes were downregulated by transfection of si-*RACGAP1* into SW1990 cells. The gene expression data were deposited in the GEO database (GEO accession number; GSE115909). Among these genes, 64 were also upregulated in PDAC clinical specimens (GEO accession number: GSE15471).

Table S1. Enriched KEGG Pathways in miR-204-5p transfectant on SW1990.

KEGG ID	Pathways	*p*-Value	No.of Genes	Genes
Kegg:04360	Axon guidance	0.00020	7	*EPHB2,EPHB6,NTN4,UNC5B,PLXNA2,EPHB3,CXCL12*
Kegg:05200	Pathways in cancer	0.03269	7	*IL6,WNT7B,COL4A6,FGFR3,PDGFRB,FN1,FGFR2*
Kegg:04810	Regulation of actin cytoskeleton	0.01382	6	*FGFR3,BDKRB1,PDGFRB,FGD3,FN1,FGFR2*
Kegg:04060	Cytokine-cytokine receptor interaction	0.03481	6	*INHBB,IL6,IL20RA,PDGFRB,CCL5,CXCL12*
Kegg:04010	MAPK signaling pathway	0.03650	6	*FGFR3,CACNG7,PDGFRB,TAOK2,DUSP22,FGFR2*
Kegg:04974	Protein digestion and absorption	0.00082	5	*SLC7A7,COL4A6,SLC1A1,DPP4,CELA3A*
Kegg:04142	Lysosome	0.00556	5	*AP1S3,AP1S2,HYAL1,M6PR,AP3M1*
Kegg:04144	Endocytosis	0.03480	5	*RAB22A,FGFR3,FOLR3,ERBB3,FGFR2*

Table S2. Enriched KEGG Pathways in si-RACGAP1 transfectant on SW1990.

KEGG ID	Pathways	*p*-Value	No.of Genes	Genes
Kegg:05145	Toxoplasmosis	0.00047	7	*IRAK4,PIK3CB,AKT3,HSPA6,CASP3,PLA2G3,PIK3CG*
Kegg:04010	MAPK signaling pathway	0.02787	7	*DUSP9,AKT3,HSPA6,DUSP5,CASP3,PDGFRB,PLA2G3*
Kegg:00240	Pyrimidine metabolism	0.00061	6	*DCK,CMPK1,NUDT2,CMPK2,DPYD,DUT*
Kegg:04620	Toll-like receptor signaling pathway	0.00094	6	*IRAK4,PIK3CB,AKT3,SPP1,IFNAR2,PIK3CG*
Kegg:04670	Leukocyte transendothelial migration	0.00168	6	*OCLN, PTPN11,PIK3CB,RASSF5,NCF2,PIK3CG*
Kegg:04650	Natural killer cell mediated cytotoxicity	0.00280	6	*PTPN11,PIK3CB,CD247,CASP3,IFNAR2,PIK3CG*
Kegg:05162	Measles	0.00340	6	*IRAK4,PIK3CB,AKT3,HSPA6,IFNAR2,PIK3CG*
Kegg:04146	Peroxisome	0.00160	5	*SCP2,SLC27A2,ACAA1,IDH1,FAR2*
Kegg:04914	Progesterone-mediated oocyte maturation	0.00275	5	*PIK3CB,CDC25C,AKT3,PIK3CG,CDK1*
Kegg:04012	ErbB signaling pathway	0.00289	5	*NRG1,NRG4,PIK3CB,AKT3,PIK3CG*
Kegg:05142	Chagas disease (American trypanosomiasis)	0.00570	5	*IRAK4,PIK3CB,AKT3,CD247,PIK3CG*
Kegg:04722	Neurotrophin signaling pathway	0.01266	5	*IRAK4,PTPN11,PIK3CB,AKT3,PIK3CG*

Kegg:04380	Osteoclast differentiation	0.01349	5	*PIK3CB,AKT3,NCF2,IFNAR2,PIK3CG*
Kegg:05160	Hepatitis C	0.01668	5	*OCLN,PIK3CB,AKT3,IFNAR2,PIK3CG*
Kegg:04910	Insulin signaling pathway	0.01668	5	*PIK3CB,AKT3,PIK3CG,PCK2,PYGL*
Kegg:04630	Jak-STAT signaling pathway	0.02845	5	*PTPN11,PIK3CB,AKT3,IFNAR2,PIK3CG*
Kegg:03320	PPAR signaling pathway	0.00773	4	*SCP2,SLC27A2,ACAA1,PCK2*
Kegg:04666	Fc gamma R-mediated phagocytosis	0.01949	4	*PIK3CB,SCIN,AKT3,PIK3CG*
Kegg:04972	Pancreatic secretion	0.02394	4	*CELA3B,RAB8A,PLA2G3,PRSS1*
Kegg:05146	Amoebiasis	0.02723	4	*SERPINB3,PIK3CB,CASP3,PIK3CG*
Kegg:00790	Folate biosynthesis	0.00036	3	*ALPPL2,ALPP,DHFR*
Kegg:00020	Citrate cycle (TCA cycle)	0.00442	3	*IDH1,PCK2,SUCLG2*
Kegg:00350	Tyrosine metabolism	0.00925	3	*ADH1A,TAT,ALDH3A1*
Kegg:05150	Staphylococcus aureus infection	0.01451	3	*CFH,FPR1,PTAFR*
Kegg:00310	Lysine degradation	0.01719	3	*SUV39H2,SUV39H1,WHSC1*
Kegg:00010	Glycolysis / Gluconeogenesis	0.03177	3	*ADH1A,PCK2,ALDH3A1*
Kegg:04920	Adipocytokine signaling pathway	0.03866	3	*PTPN11,AKT3,PCK2*
Kegg:04115	p53 signaling pathway	0.03866	3	*CD82,CASP3,CDK1*

Table S3. EMT related genes regulated by si-RACGAP1 and miR-204-5p.

Entrez Gene ID	GeneSymbol	GeneName	SW1990 si-*RACGAP1* Trabsfectant FC(log2)	SW1990 *miR-204-5p* Trabsfectant FC(log2)
1009	*CDH11*	cadherin 11, type 2, OB-cadherin (osteoblast)	1.37100	−1.53382
6615	*SNAI1*	snail family zinc finger 1	1.21199	−1.06168
6591	*SNAI2*	snail family zinc finger 2	−1.26224	−1.23785
333929	*SNAI3*	snail family zinc finger 3	−2.86950	−1.05288
2113	*ETS1*	v-ets avian erythroblastosis virus E26 oncogene homolog 1	1.39537	1.39185
6935	*ZEB1*	zinc finger E-box binding homeobox 1	−1.32748	1.10573
7291	*TWIST1*	twist family bHLH transcription factor 1	−1.53565	−1.54288
7431	*VIM*	vimentin	−2.47805	1.21032
2335	*FN1*	fibronectin 1	−1.20706	−2.03232
1000	*CDH2*	cadherin 2, type 1, N-cadherin (neuronal)	1.36821	−1.18938

Table S4. MET related genes regulated by si-RACGAP1 and miR-204-5p.

Entrez Gene ID	GeneSymbol	GeneName	SW1990 si-*RACGAP1* Trabsfectant FC(log2)	SW1990 *miR-204-5p* Trabsfectant FC(log2)
999	*CDH1*	cadherin 1, type 1, E-cadherin (epithelial)	−1.18868	−1.66021
7082	*TJP1*	tight junction protein 1	1.23079	1.24608
6382	*SDC1*	syndecan 1	−1.03295	−1.00811
6383	*SDC2*	syndecan 2	−1.86755	−1.55283
9672	*SDC3*	syndecan 3	1.32986	−1.38467
6385	*SDC4*	syndecan 4	1.06226	−1.19386
1282	*COL4A1*	collagen, type IV, alpha 1	1.14062	−1.61619
4582	*MUC1*	mucin 1, cell surface associated	−1.02071	−1.46347
1832	*DSP*	desmoplakin	−1.28556	−1.03749

© 2019 by the authors. Licensee MDPI, Basel, Switzerland. This article is an open access article distributed under the terms and conditions of the Creative Commons Attribution (CC BY) license (http://creativecommons.org/licenses/by/4.0/).
